# The Association between Health Beliefs and Fall-Related Behaviors and Its Implication for Fall Intervention among Chinese Elderly

**DOI:** 10.3390/ijerph16234774

**Published:** 2019-11-28

**Authors:** Fenfen Li, Deding Zhou, Yue Chen, Yan Yu, Ning Gao, Juanjuan Peng, Shumei Wang

**Affiliations:** 1 School of Public Health, Key Laboratory of Public Health Safety, Ministry of Education, Fudan University, Shanghai 200032, China; liff17@fudan.edu.cn; 2 Shanghai Municipal Center for Disease Control and Prevention, Shanghai 200336, China; zhoudeding@scdc.sh.cn (D.Z.); yuyan@scdc.sh.cn (Y.Y.); gaoning@scdc.sh.cn (N.G.); 3 School of Epidemiology and Public Health, Faculty of Medicine, University of Ottawa, Ottawa, ON K1G 5Z3, Canada; ychen@uottawa.ca

**Keywords:** falls, the elderly, health belief, risk behavior, association

## Abstract

To apply the Health Belief Model (HBM) to fall prevention of the elderly and estimate fall health beliefs and their relationships with fall-related behaviors, a citywide cross-sectional study was conducted among people aged 60 years or over in 13 out of 16 districts in Shanghai, China, in September 2018. A total of 5833 participants were investigated. Of this, 43.4% were male; 48.8% were aged 60–69; 18.1% were uneducated; and 50.3% were living in urban areas. People who were older, less educated, living in rural areas generally had lower scores in the 7 HBM dimensions and also had lower proportions of fall risk-reduction behaviors, except that the less educated elderly were more likely to participate in exercise and training and the rural elderly were more likely to check house environment and participate in exercise and training (*p* < 0.001). The HBM dimensions were generally positively correlated with the risk-reduction behaviors except that “perceived severity” was negatively correlated with four risk-reduction behaviors and behavior number, “cues to action” was negatively correlated with purchasing shoes, and “perceived benefits” was negatively correlated with participating in exercise activities and fall prevention training (*p* < 0.05). When HBM is applied in the field of fall prevention, the interpretation of the results of each dimension has its characteristics in the fields of injury research. Fall prevention strategies should focus on improving the health beliefs and behaviors in those who were older, less educated and living in rural areas, implementing different levels of fall prevention activities to meet different needs, improving the accessibility and applicability of related resources, and raising the organizational level of related fall prevention activities.

## 1. Introduction

Fall injury of the elderly has become a severe public health problem because of aging populations [1] and disease spectrum shift [2]. Fall is the leading cause of death among all types of injuries in elderly people [3]. China is facing a huge challenge of an aging population, and there were approximately 241 million (17.3%) people aged 60 years or over at the end of 2017 [4], with a larger proportion in mega-cities, e.g., 33.2% in Shanghai [5]. It was reported that 60%–75% of the falls caused injuries and that 6%–8% of the fall-related injuries were fractures [6]. Fall injuries accounted for more than half of all types of injuries [7]. Fall injury has become one of the top 15 causes of death in China since 2013 [8]. The age-standardized death rate from falls increased by 12.0% from 7.89 in 1990 to 8.52 per 100,000 people in 2017 [9]. Outcomes also include various disabilities and hospitalization [10], causing economic loss and social burden [2,3].

Therefore, preventing falls should be a priority of public health strategies for the elderly. The factors affecting falls of the elderly people are complicated, including social demographic factors, physical function status, mental states, chronic diseases, medication usage, lifestyle factors, and living environment [11,12,13,14]. It is crucial for fall prevention to correctly recognize these risk factors and then take appropriate measures, such as using appropriate auxiliary equipment, wearing suitable shoes and clothes, creating a safe home environment, and exercising regularly and safely [3,11,12]. It is also important for medical professionals to evaluate fall-related risk awareness, the perceptions and behaviors of the elderly people, to identify fall prevention needs and to find solutions.

The Health Belief Model (HBM) is a classic and widely used psychological theory in health science. It is believed that individual’s behaviors are the results of psychological activities, and the most direct psychological activities that determine people to take certain behaviors are perception, attitude, and belief [15]. The HBM defines the key factors that influence health behaviors as an individual’s perceived threat to sickness or disease (perceived susceptibility), belief of adverse consequence (perceived severity), potential positive benefits of action (perceived benefits), perceived barriers to action, exposure to factors that prompt action (cues to action), health motivation, and confidence in ability to succeed (self-efficacy) [16,17]. Psychological activities were divided into several factors to synthetically explain why people take or not take a specific action. The HBM has been successfully applied in health education and health promotion for explaining and promoting preventive health behaviors [18,19,20,21], including the field of injury prevention [22,23,24]. The purpose of this study was to apply the HBM to fall prevention of the elderly by estimating fall health beliefs and the relationship with fall-related behaviors order to explore the possibilities to intervene falls among elderly people.

## 2. Materials and Methods

### 2.1. Study Design and Participants

This was a cross-sectional study conducted among men and women aged 60 or over from 13 out of 16 districts in Shanghai, China, in 2018. The participants were selected using multi-stage sampling. Of the 13 districts, 7 were urban districts and 6 were rural districts. In each district, one community was conveniently sampled. A community was defined as a sub-district in an urban district or a town in a rural district according to the administrative division in China. In each community, a neighborhood or a village was randomly selected. In the neighborhood or village, those who were not able to walk either with or without assistive devices or with severe medical problems were excluded. All eligible elderly people were informed about the purposes and contents of the investigation and the inclusion criteria. The elderly people were gathered in the Community Health Service Center, and the investigation was conducted by well-trained doctors in the center until the sample size met the requirement. At least 430 elderly adults were recruited from each community, which was calculated based on fall incidence in Shanghai. A total of 5833 participants were investigated, including information on demographic factors, fall health beliefs and fall-related behaviors. All procedures performed in the study involving human participants were in accordance with the ethical standards of the Ethics Committee of Fudan University (International registration number: IRB00002408&FWA00002399). Informed consent was obtained from each participant.

### 2.2. Fall-Related HBM Scale Development and Assessment

Following an investigation of falls risk factors and epidemiologic characteristics in Shanghai, the review of literature and consultation with researchers and health professionals, a short self-administered questionnaire was designed to capture health belief indicators related to falls among the elderly. The questionnaire was composed of 7 dimensions with 26 items according to the HBM (see Table 1 for dimensions and items). A 5-point Likert scale was used to rate the items with 1-5 mark for ‘strongly disagree’, ‘disagree’, ‘neutral’, ‘agree’, and ‘strongly agree’. The 5 items of perceived barriers were reverse coded so that the effect was in the direction hypothesized by the HBM theory. The questionnaire was further evaluated and modified by experts in the field of epidemiology, injury prevention mapping, health education, and fall prevention professionals for its content validity and clarity.

**Figure 1:**
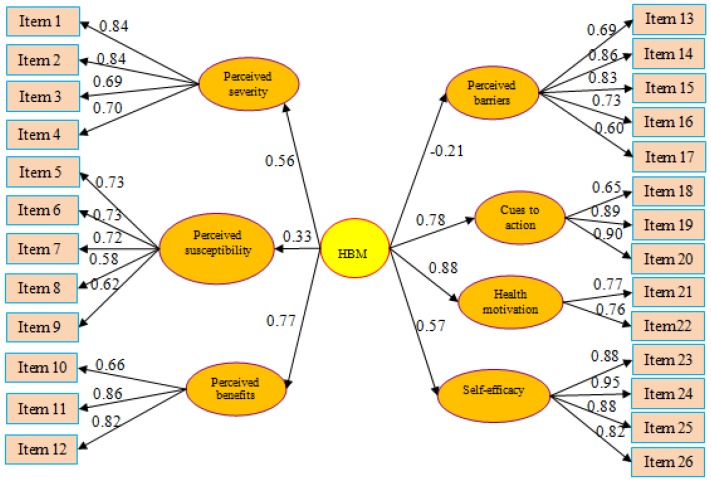
Health Belief Model (HBM) path diagram and the standardized path coefficient.

Item analysis, item-scale correlation, and correlation matrix were performed. These include a frequency table of responses for each of the 26 items, uncorrected item-scale correlations, and the correlation matrix among the 26 items (Appendix A). For the 26 items, 68% (88 out of 130) of the responses fell in the range of 5%–95%. The uncorrected item-scale correlations ranged from 0.57–0.86. Within each of the 7 dimensions, items were significantly correlated; and the 26 items were significantly correlated with each other (*p* < 0.05). The reliability score of the whole HBM scale by Cronbach’s alpha test was 0.91 and for the HBM dimensions was between 0.75 and 0.93 (Table 1). A structural validity test of confirmatory factor analysis was made by using AMOS 23.0 and reported acceptable fit statistics: Root Mean Square Error of Approximation = 0.03 (≤0.08 indicates good fit), Goodness of Fit Index = 0.95 (≥0.90 indicates good fit), Adjusted Goodness of Fit Index = 0.94 (≥0.90 indicates good fit), Comparative Fit Index = 0.97 (≥0.90 indicates good fit). Figure 1 shows the factor structure of the HBM and the standardized path coefficient. Confirmatory Factor Analysis Weighted Score method was used to calculate the weighted HBM dimension scores with a higher score representing a better fall heath belief [22,25,26].

### 2.3. Fall-Related Behaviors

With the HBM questionnaire, seven fall-related behaviors of the elderly over the past 12 months were investigated by asking the following questions: (1)“Will you have a trial before purchasing crutches or other walking aids?”; (2)“will you have optometry check before purchasing presbyopic glasses?”; (3)“will you have hearing tests before purchasing hearing-aid”; (4)“did you try your shoes before purchasing?”; (5)“did you often check your house environment to prevention fall?”; (6)“did you participate in the organized exercise activities to prevention fall?”; and (7)“did you participate in the organized training on fall prevention for the elderly?”. Each question has 2 options of “yes” and “no”, with “yes” representing a correct behavior and “no” representing a risk behavior. The total “yes” number of the 7 behavior questions was accumulated as risk-reduction behavior number, with higher number representing better behavior. A “risk-reduction behavior group” was defined as those who chose “yes” for each fall-related behavior, and a “risk behavior group” was defined as those who chose “no” for each fall-related behavior.

### 2.4. Statistical Analysis

Statistical analyses were performed with AMOS 23.0 and SPSS V21.0 software. Confirmatory factor analysis was performed by using AMOS 23.0 to test the structural validity of the HBM scale. Cronbach’s alpha test was performed to analyze the reliability of the overall HBM scale and HBM dimensions. Confirmatory Factor Analysis Weighted Score method was used to calculate the weighted HBM dimension scores [22,26]. Descriptive statistics such as means and proportions were used to describe the distributions of demographic characteristics, HBM dimension scores, and fall-related behaviors. Heat plot was used to display the fall risk-reduction behavior proportions according to demographic variables. Chi-square tests were performed to compare fall-related behaviors according to demographic variables and the demographic characteristics between the risk-reduction behavior group and the risk behavior group. Independent-samples t-tests were performed to compare HBM dimension scores according to demographic variables and between the risk-reduction behavior and risk behavior groups. Multivariate logistic regression analysis was conducted to analyze the association between HBM dimensions and fall-related risk-reduction behaviors. A generalized linear model was conducted to analyze the association between HBM dimensions and fall-related behavior number. Odds ratios (ORs) and 95% confidence intervals (CIs) were calculated. An alpha level of less than 5% was considered to be statistically significant.

## 3. Results

Of the 5833 participants, 2531 (43.4%) were male; 2847 (48.8%) aged 60 to 69; 1056 (18.1%) were uneducated; and 2934 (50.3%) were living in urban areas (Table 2). The overall HBM score was 36.88 ± 4.47. Scores of the 7 HBM dimensions ranged from 5.99 ± 0.88 (“health motivation”) to 14.00 ± 1.66 (“perceived susceptibility”). Compared with men, women scored higher on “health motivation”, and “self-efficacy”. People who were older, less educated, living in rural areas generally scored lower (*p* < 0.05) (Table 3).

**Figure 2:**
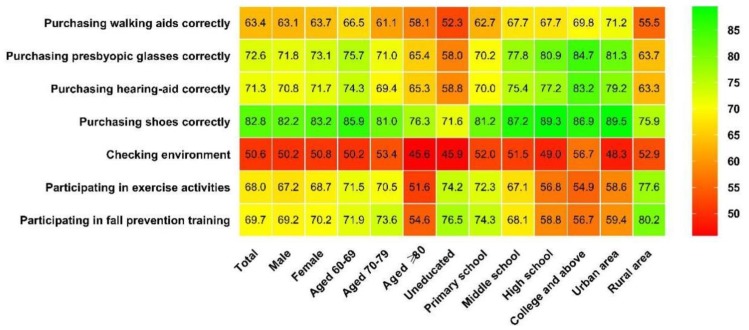
Heat plot of fall risk-reduction behavior proportions and associated demographic variables.

Figure 2 shows the heat plot of fall risk-reduction behaviors in associations with demographic factors. The proportion of risk-reduction behavior of checking environment was the lowest, followed by purchasing walking aids correctly. Women tended to have higher proportions of all the risk-reduction behaviors compared with men, but the gender differences were not statistically significant (*p* > 0.05). The proportions of risk-reduction behaviors decreased with age (*p* < 0.001). Education level was negatively associated with participating in exercise and training, but was positively associated with other risk-reduction behaviors (*p* < 0.001). Compared with rural elderly people, urban elderly were more likely to correctly buy walking aids, presbyopic glasses, hearing-aid and proper shoes, but were less likely to check house environment and participate in exercise and training (*p* < 0.001).

**Figure 3:**
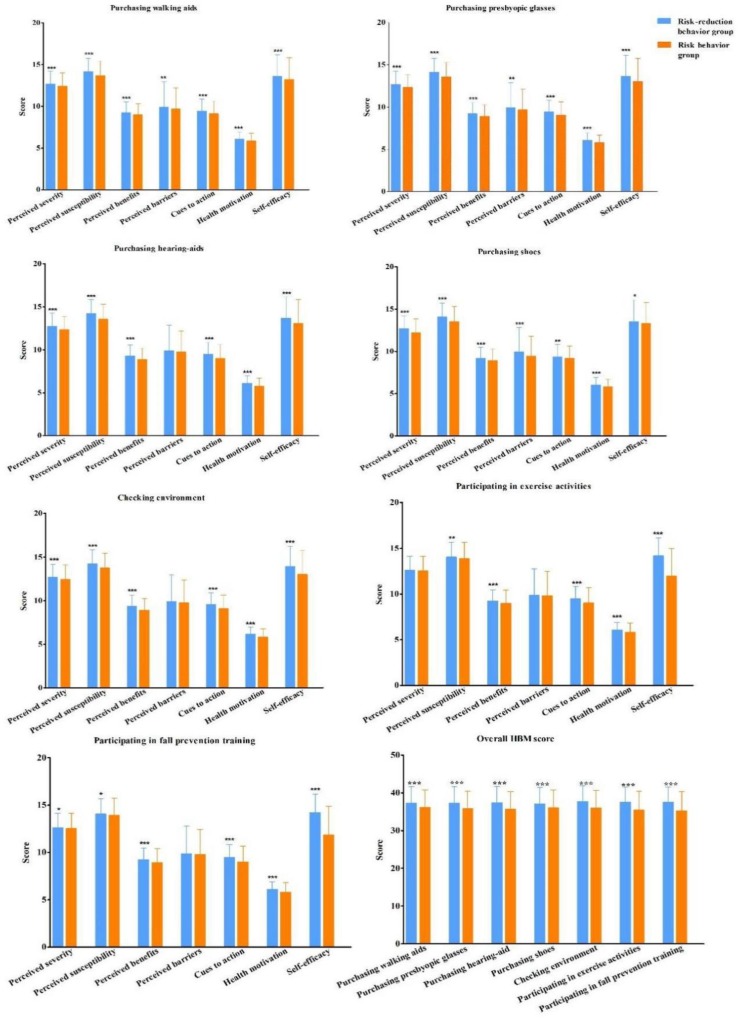
Comparison of HBM Scores between risk behavior group and risk-reduction behavior group Note: *** *p* < 0.001. ** *p* < 0.01. * *p* < 0.05.

Figure 3 shows the HBM dimension scores in the risk behavior group and risk-reduction behavior group. For all the seven fall-related behaviors, HBM dimension scores and the overall HBM scores were higher in risk-reduction behavior group than in risk behavior group (*p* < 0.05), except for “perceived barriers” in purchasing hearing-aid correctly, checking environment, participating in exercise activities, and participating in fall prevention training.

Table 4 shows the demographic characteristics of the risk-reduction behavior group and the risk behavior group. Compared with the risk behavior group, the risk-reduction behavior group had larger proportions of women and younger participants, and there was no significant gender difference between the two groups. In the risk-reduction behavior group, participants with higher education and urban elderly were more likely to have the behaviors of purchasing walking aids, purchasing presbyopic glasses, purchasing hearing-aid, and purchasing shoes, but were less likely to have the behaviors of checking environment, participating in exercise activities, and participating in fall prevention training.

**Figure 4:**
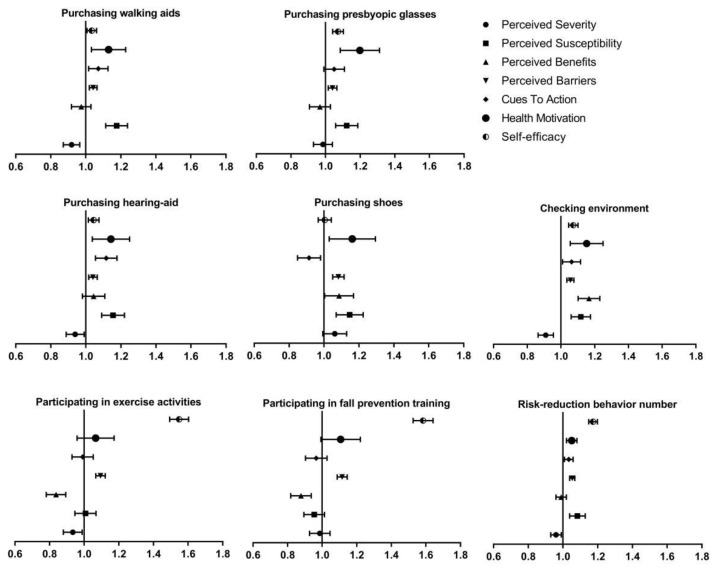
The association between HBM dimensions and fall-related risk-reduction behaviors and behavior number. Note: The results were expressed as odds ratios (OR) and 95% confidence interval (CI). Adjusted demographic variables of gender, age, education and living place.

The effect of HBM dimensions on the seven fall-related risk-reduction behaviors and behavior number was shown in Figure 4. The HBM dimensions were generally positively correlated with the risk-reduction behaviors and behavior number except that “perceived severity” was negatively correlated with four risk-reduction behaviors and behavior number, “cues to action” was negatively correlated with purchasing shoes, and “perceived benefits” was negatively correlated with participating in exercise activities and fall prevention training.

## 4. Discussion

This study used HBM to prevent falls in the elderly. The reliability and validity of the HBM scale is good. In the programs for the elderly fall prevention health education, HBM has been used to design health education content and is used to assess the effectiveness of these educational tools. However, the reliability and validity of these tools have not been verified, or the relevant information is not described in details [27,28]. In addition, the application of HBM to fall prevention in different elderly populations who have different demographic characteristics is rare.

The HBM dimension scores of risk-reduction behavior groups were higher than those of the risk behavior groups. The elderly’s risk-reduction behaviors and risk-reduction behavior number were generally positively correlated with the HBM dimensions score. This is consistent with the findings of HBM theory in the study of other diseases [19,20].

However, “perceived severity” is negatively correlated with four risk-reduction behaviors and the risk-reduction behavior number. This may be due to the fact that when the elderly are worried about falls, some of their fall-prevention actions are constrained. For example, they will do less exercise. Generally, a higher perceived severity score indicates a higher level of awareness towards the severity of disease or untreated situation, and a greater likelihood of behavior improvement due to the fear of serious consequences. But in this case, it is the opposite. This may also be the difference between injury prevention research and research of other diseases, such as chronic diseases.

“Cues to action” has a negative effect on the purchase of correct shoes, probably due to the rapid development of online shopping in China and the limited number of physical stores [29]. TV shopping, mobile shopping, and online shopping may all affect shopping behavior of the elderly. On the one hand, the proportion of elderly who do online shopping is getting higher and higher. On the other hand, children of the elderly shop for the elderly mainly using the internet. Therefore, compared with the traditional physical store shopping, the try-on behavior is less and less.

The negative impact of “perceived benefits” on health promotion behaviors is rare. But our results indicate that “perceived benefits” are negatively correlated with participation in physical activities and fall prevention training. Since these two activities are closely related to community resources and organizational conditions, the factors including accessibility and applicability of exercise facilities and places, the faculty/content/form of training activities can influence the elderly’s participation in physical activity and fall prevention training. Even if the elderly have a high “perceived benefits” score, it is possible that the relevant activities and training provided by the community cannot meet the actual needs of the elderly, thus the behavior does not correspond to the perceived benefit scores. The findings also provide a reference for future improvements in community fall injury interventions.

Education level represents knowledge, economic status and social status to a certain extent. This study showed that education level is positively correlated with fall-related health beliefs and risk-reduction behaviors in general. However, we also found that elderly with higher education had poorer “self-efficacy”, and were less likely to participate in exercise and training. Higher-educated elderly have significantly higher awareness of “perceived severity”, “perceived susceptibility” and “perceived barriers” than lower-educated elderly, as the one who knows nothing fears nothing. This may also lead to their lack of confidence in the implementation of fall intervention measures. Higher-educated elderly have higher requirements for related activities and training. Some of the current activities may not meet their expectations and cannot provide corresponding services for the elderly with different levels of needs. Generally speaking, except for the above two points, lower-educated elderly had poorer fall prevention health beliefs and behaviors, which need more attention.

There are differences in health beliefs and fall-related behaviors between urban and rural areas. Except for “cues to action” and “self-efficacy”, urban elderly have a higher score in the other five HBM dimensions. This may be due to the urban-rural differences in daily life, knowledge levels, and medical services barriers. Compared with urban elderly, the rural elderly’s purchasing behaviors of fall prevention related equipment and daily necessities need to be improved, especially for those who cannot purchase related equipment, suitable shoes and other life necessities due to lack of correct purchasing route and low purchasing power. Since the “self-efficacy” score of rural elderly is higher than that of urban elderly, providing appropriate resources and guidance may improve rural elderly’s behaviors quickly and effectively.

This study was conducted in 13 districts in Shanghai and was a sub-project of Shanghai Elderly Fall Prevention Project. Given the big coverage and sample size, communities were conveniently selected to ensure feasibility and sustainability. Therefore, selection bias may exist. In addition, Delphi consultation was not conducted during the development of fall HBM scale, and that is why content validity was not available in this study. However, several rounds of experts’ consultation meetings in related fields were held apart from the literature review. The Cronbach coefficient and fit statistics of confirmatory factor analysis also showed that the reliability and validity of the HBM scale were acceptable in this study.

## 5. Conclusions

This study used HBM to promote fall prevention in different elderly populations who have different demographic characteristics. When HBM is applied in the field of fall prevention, the interpretation of the results of each dimension has characteristics in the fields of injury research. The focus of fall prevention strategies is to improve the health beliefs and behaviors in those who were older, less educated and living in rural areas, implementing different levels of fall prevention activities to meet different needs, improving the accessibility and applicability of related resources, and raising the organizational level of related fall prevention activities.

## Figures and Tables

**Table 1:** The fall-related health belief scale.

Dimensions	Item Number	Items	Cronbach α
Perceived severity (Belief about how serious a condition and its sequelae are)	1	Fall in the elderly is a very serious problem.	0.86
	2	Fall in the elderly can cause fractures, disability, and even death.	
	3	Fall in the elderly can change psychology and cause fear of fall.	
	4	Fall in the elderly can increase the burden on the family.	
Perceived susceptibility (Belief about the chances of experiencing a risk or getting a condition or disease)	5	The elderly people are prone to fall.	0.87
	6	Insecurities in the home and community can easily lead to falls, such as slippery floors, aisle debris, etc.	
	7	Some bad habits can cause falls, including unsuitable dressing and shoes, not using handrails, etc.	
	8	Unhealthy mental states can cause falls, such as depression.	
	9	Many chronic diseases and organ hypofunction can cause fall.	
Perceived benefits (Belief in efficacy of the advised action to reduce risk or seriousness of impact)	10	Falls of the elderly is preventable with right methods.	0.85
	11	It will decrease the risk of falls if I can change the insecurities in the home environment.	
	12	It will decrease the risk of falls if I can change my bad habits.	
Perceived barriers (Belief about the tangible and psychological costs of the advised action)	13	I know some habits are bad, but it’s hard for me to make changes.	0.86
	14	It’s hard for me to change some of the insecurities in my home environment.	
	15	It’s hard for me to determine the risk factor of falls.	
	16	It is difficult for me to adhere to the treatment of chronic diseases that affect falls, such as hypertension.	
	17	It is expensive to prevent falls, such as installing handrails.	
Cues to action (Strategies to activate readiness and promote awareness)	18	Fall prevention information on TV commercials and publication propaganda has an impact on me.	0.87
	19	Fall experiences from family members and friends have an impact on me.	
	20	Views of family members and friends on fall hazards and prevention have an impact on me.	
Health motivation (Awareness of prevention of a risk, a condition or disease)	21	I usually value my safety.	0.75
	22	I usually take the initiative to acquire injury prevention knowledge.	
Self-efficacy (Confidence in one’s ability to take action)	23	I am willing to participate in falls prevention activities.	0.93
	24	I can complete the task assigned to me while participating in falls prevention activities.	
	25	I can make up my mind to correct my bad habits while participating in falls prevention activities.	
	26	I can change the insecurities in the home environment while participating in falls prevention activities.	
Overall			0.91

**Table 2:** Demographic characteristics of the 5833 study participants.

Variables	N	%
Gender		
Male	2531	43.4
Female	3302	56.6
Age (years)		
60–69	2847	48.8
70–79	2010	34.5
≥80	976	16.7
Education		
Uneducated	1056	18.1
Primary school	1842	31.6
Middle school	1857	31.8
High school	810	13.9
College and above	268	4.6
Area of residence		
Urban area	2934	50.3
Rural area	2899	49.7

**Table 3:** HBM dimensions scores and associated demographic variables.

Demographic Variable	Perceived Severity	Perceived Susceptibility	Perceived Benefits	Perceived Barriers	Cues to Action	Health Motivation	Self-Efficacy	Overall HBM Score
Total	12.59 ± 1.55	14.00 ± 1.66	9.15 ± 1.30	9.85 ± 2.83	9.32 ± 1.47	5.99 ± 0.88	13.48 ± 2.57	36.88 ± 4.47
Gender								
Male (Ref)	12.55 ± 1.56	13.97 ± 1.66	9.12 ± 1.28	9.83 ± 2.80	9.30 ± 1.47	5.97 ± 0.88	13.41 ± 2.55	36.75 ± 4.46
Female	12.62 ± 1.54	14.02 ± 1.67	9.17 ± 1.32	9.86 ± 2.85	9.34 ± 1.47	6.02 ± 0.89 *	13.53 ± 2.58 *	36.98 ± 4.48
Age (years)								
60–69 (Ref)	12.62 ± 1.56	14.04 ± 1.64	9.21 ± 1.31	9.98 ± 2.92	9.34 ± 1.47	6.01 ± 0.88	13.66 ± 2.49	37.06 ± 4.39
70–79	12.59 ± 1.52	13.98 ± 1.66	9.16 ± 1.28	9.74 ± 2.77 **	9.35 ± 1.46	6.02 ± 0.88	13.52 ± 2.56	36.97 ± 4.46
≥80	12.48 ± 1.58 *	13.90 ± 1.74 *	8.94 ± 1.29 ***	9.60 ± 2.60 ***	9.21 ± 1.48 *	5.88 ± 0.89 ***	12.79 ± 2.73 ***	36.09 ± 4.66 ***
Education								
Uneducated (Ref)	12.50 ± 1.52	13.86 ± 1.65	9.10 ± 1.23	9.32 ± 2.59	9.37 ± 1.44	5.96 ± 0.85	13.55 ± 2.44	36.90 ± 4.47
Primary school	12.42 ± 1.52	13.81 ± 1.64	9.12 ± 1.26	9.62 ± 2.65 *	9.27 ± 1.39	5.93 ± 0.86	13.48 ± 2.47	36.65 ± 4.36
Middle school	12.77 ± 1.57 ***	14.16 ± 1.68 ***	9.19 ± 1.38	10.16 ± 3.00 ***	9.39 ± 1.51	6.04 ± 0.89 *	13.62 ± 2.59	37.18 ± 4.55
High school	12.67 ± 1.57	14.19 ± 1.62 ***	9.17 ± 1.34	10.04 ± 2.94 ***	9.22 ± 1.62	6.03 ± 0.94	13.14 ± 2.85 **	36.72 ± 4.61
College and above	12.59 ± 1.56	14.16 ± 1.69 *	9.21 ± 1.23	10.69 ± 2.88 ***	9.33 ± 1.43	6.13 ± 0.87 *	13.18 ± 2.62 **	36.75 ± 4.13
Living place								
Urban area (Ref)	12.74 ± 1.58	14.23 ± 1.69	9.20 ± 1.40	10.19 ± 3.00	9.34 ± 1.57	6.03 ± 0.94	13.21 ± 2.84	36.90 ± 4.77
Rural area	12.44 ± 1.50 ***	13.77 ± 1.61 ***	9.09 ± 1.19 ***	9.50 ± 2.59 ***	9.31 ± 1.36	5.96 ± 0.82 ***	13.75 ± 2.23 ***	36.85 ± 4.14

Note: The results were expressed as Mean ± SD; *** *p* < 0.001; ** *p* < 0.01; * *p* < 0.05.

**Table 4:** Demographic characteristics of the risk-reduction behavior group and risk behavior group.

Demographic Variable	Purchasing Walking Aids			Purchasing Presbyopic Glasses			Purchasing Hearing-Aid			Purchasing Shoes			Checking Environment			Participating in Exercise Activities			Participating in Fall Prevention Training		
	Risk-Reduction Behavior Group	Risk Behavior Group	*p*-Value	Risk-Reduction Behavior Group	Risk Behavior Group	*p*-Value	Risk-Reduction Behavior Group	Risk Behavior Group	*p*-Value	Risk-Reduction Behavior Group	Risk Behavior Group	*p*-Value	Risk-Reduction Behavior Group	Risk Behavior Group	*p*-Value	Risk-Reduction Behavior Group	Risk Behavior Group	*p*-Value	Risk-Reduction Behavior Group	Risk Behavior Group	*p*-Value
Gender			0.620			0.253			0.445			0.346			0.612			0.209			0.416
Male	1596(43.1)	935(43.8)		1817(42.9)	714(44.6)		1792(43.1)	739(44.2)		2081(43.1)	450(44.7)		1270(43.1)	1261(43.7)		1700(42.8)	831(44.6)		1751(43.0)	780(44.2)	
Female	2103(56.9)	1199(56.2)		2415(57.1)	887(55.4)		2368(56.9)	934(55.8)		2746(56.9)	556(55.3)		1679(56.9)	1623(56.3)		2269(57.2)	1033(55.4)		2317(57.0)	985(55.8)	
Age (year)			<0.001			<0.001			<0.001			<0.001			<0.001			<0.001			<0.001
60–69	1997(54.0)	1004(47.0)		2272(53.7)	729(45.5)		2231(53.6)	770(46.0)		2577(53.4)	424(42.1)		1507(51.1)	1494(51.8)		2145(54.0)	856(45.9)		2157(53.0)	844(47.8)	
70–79	1171(31.7)	747(35.0)		1362(32.2)	556(34.7)		1332(32.0)	586(35.0)		1553(32.2)	365(36.3)		1025(34.8)	893(31.0)		1352(34.1)	566(30.4)		1412(34.7)	506(28.7)	
≥80	531(14.4)	383(17.9)		598(14.1)	316(19.7)		597(14.4)	317(18.9)		697(14.4)	217(21.6)		417(14.1)	497(17.2)		472(11.9)	442(23.7)		499(12.3)	415(23.5)	
Education			<0.001			<0.001			<0.001			<0.001			0.003			<0.001			<0.001
Uneducated	552(14.9)	504(23.6)		612(14.5)	444(27.7)		621(14.9)	435(26.0)		756(15.7)	300(29.8)		485(16.4)	571(19.8)		784(19.8)	272(14.6)		808(19.9)	248(14.1)	
Primary school	1155(31.2)	687(32.2)		1293(30.6)	549(34.3)		1290(31.0)	552(33.0)		1496(31.0)	346(34.4)		958(32.5)	884(30.7)		1332(33.6)	510(27.4)		1368(33.6)	474(26.9)	
Middle school	1257(34.0)	600(28.1)		1445(34.1)	412(25.7)		1401(33.7)	456(27.3)		1619(33.5)	238(23.7)		957(32.5)	900(31.2)		1246(31.4)	611(32.8)		1264(31.1)	593(33.6)	
High school	548(14.8)	262(12.3)		655(15.5)	155(9.7)		625(15.0)	185(11.1)		723(15.0)	87(8.6)		397(13.5)	413(14.3)		460(11.6)	350(18.8)		476(11.7)	334(18.9)	
College and above	187(5.1)	81(3.8)		227(5.4)	41(2.6)		223(5.4)	45(2.7)		233(4.8)	35(3.5)		152(5.2)	116(4.0)		147(3.7)	121(6.5)		152(3.7)	116(6.6)	
Living place			<0.001			<0.001			<0.001			<0.001			<0.001			<0.001			<0.001
Urban area	2089(56.5)	845(39.6)		2385(56.4)	549(34.3)		2324(55.9)	610(36.5)		2626(54.4)	308(30.6)		1416(48.0)	1518(52.6)		1719(43.3)	1215(65.2)		1743(42.8)	1191(67.5)	
Rural area	1610(43.5)	1289(60.4)		1847(43.6)	1052(65.7)		1836(44.1)	1063(63.5)		2201(45.6)	698(69.4)		1533(52.0)	1366(47.4)		2250(56.7)	649(34.8)		2325(57.2)	574(32.5)	

## Appendix A

**Table A1:** Item distribution, item–scale correlation, and correlation matrix.

Item	Distribution of Grades					Uncorrected Item-Scale Correlation	Correlation Matrix																									
	(% of Each Grade)																															
	1	2	3	4	5		Item 1	Item 2	Item 3	Item 4	Item 5	Item 6	Item 7	Item 8	Item 9	Item 10	Item 11	Item 12	Item 13	Item 14	Item 15	Item 16	Item 17	Item 18	Item 19	Item 20	Item 21	Item 22	Item 23	Item 24	Item 25	Item 26
Item1	0.22	0.96	9.03	67.7	22.08	0.76 **	1																									
Item2	0.15	0.74	8.33	68.37	22.41	0.76 **	0.76 **	1																								
Item3	0.31	2.85	11.98	67	17.86	0.69 **	0.59 **	0.59 **	1																							
Item4	0.14	0.84	7.03	65.22	26.78	0.72 **	0.58 **	0.60 **	0.57 **	1																						
Item5	0.15	1.11	9.99	67.02	21.72	0.68 **	0.57 **	0.56 **	0.54 **	0.64 **	1																					
Item6	0.22	0.62	8.81	68.59	21.76	0.71 **	0.52 **	0.52 **	0.50 **	0.57 **	0.62 **	1																				
Item7	0.24	0.91	10.35	69.38	19.12	0.69 **	0.51 **	0.51 **	0.53 **	0.51 **	0.59 **	0.75 **	1																			
Item8	0.46	3.33	19.42	65.23	11.55	0.63 **	0.40 **	0.37 **	0.43 **	0.33 **	0.39 **	0.43 **	0.47 **	1																		
Item9	0.38	1.77	13.46	69.02	15.38	0.65 **	0.45 **	0.44 **	0.42 **	0.42 **	0.44 **	0.50 **	0.50 **	0.67 **	1																	
Item10	0.34	2.09	14.35	68.42	14.8	0.71 **	0.37 **	0.37 **	0.34 **	0.33 **	0.39 **	0.39 **	0.41 **	0.43 **	0.45 **	1																
Item11	0.31	2.14	17.44	67	13.12	0.82 **	0.34 **	0.34 **	0.33 **	0.32 **	0.37 **	0.40 **	0.44 **	0.44 **	0.42 **	0.61 **	1															
Item12	0.26	2.49	19.08	65.11	13.06	0.81 **	0.33 **	0.33 **	0.32 **	0.29 **	0.34 **	0.37 **	0.44 **	0.45 **	0.40 **	0.56 **	0.76 **	1														
Item13	7.27	50.44	26.8	13.85	1.65	0.62 **	−0.12 **	−0.13 **	−0.19 **	−0.11 **	−0.17 **	−0.16 **	−0.15 **	−0.28 **	−0.31 **	−0.18 **	−0.18 **	−0.15 **	1													
Item14	5.61	44.52	26.88	20.42	2.57	0.76 **	−0.06 **	−0.06 **	−0.12 **	0.01	−0.06 **	−0.04 **	−0.09 **	−0.18 **	−0.15 **	−0.13 **	−0.11 **	−0.13 **	0.63 **	1												
Item15	6.07	47.83	25.9	18.1	2.09	0.74 **	−0.06 **	−0.08 **	−0.10 **	−0.01	−0.05 **	−0.03 **	−0.07 **	−0.19 **	−0.14 **	−0.13 **	−0.13 **	−0.14 **	0.54 **	0.72 **	1											
Item16	5.95	39.67	22.73	26.33	5.31	0.71 **	0.01	0.01	−0.08 **	0.06 **	0.01	0.03 *	−0.03 *	−0.18 **	−0.08 **	−0.12 **	−0.10 **	−0.13 **	0.46 **	0.62 **	0.64 **	1										
Item17	8.88	47.73	25.65	15.79	1.95	0.57 **	−0.11 **	−0.10 **	−0.15 **	−0.09 **	−0.16 **	−0.13 **	−0.12 **	−0.23 **	−0.20 **	−0.27 **	−0.18 **	−0.15 **	0.42 **	0.49 **	0.49 **	0.52 **	1									
Item18	0.55	4.41	24.12	61.8	9.12	0.73 **	0.26 **	0.24 **	0.27 **	0.21 **	0.27 **	0.30 **	0.33 **	0.38 **	0.35 **	0.36 **	0.43 **	0.43 **	−0.17 **	−0.14 **	−0.15 **	−0.17 **	−0.20 **	1								
Item19	0.63	3.07	18.69	65.68	11.93	0.82 **	0.31 **	0.32 **	0.33 **	0.32 **	0.35 **	0.36 **	0.38 **	0.40 **	0.40 **	0.40 **	0.43 **	0.43 **	−0.20 **	−0.15 **	−0.16 **	−0.14 **	−0.19 **	0.61 **	1							
Item20	0.55	2.83	19.58	65.58	11.47	0.82 **	0.31 **	0.32 **	0.33 **	0.30 **	0.34 **	0.34 **	0.39 **	0.41 **	0.40 **	0.37 **	0.44 **	0.44 **	−0.18 **	−0.15 **	−0.16 **	−0.14 **	−0.17 **	0.59 **	0.80 **	1						
Item21	0.31	0.63	12.39	69.74	16.92	0.83 **	0.39 **	0.38 **	0.33 **	0.39 **	0.39 **	0.40 **	0.40 **	0.38 **	0.43 **	0.45 **	0.46 **	0.43 **	−0.13 **	−0.03 **	−0.06 **	−0.03 **	−0.13 **	0.42 **	0.52 **	0.54 **	1					
Item22	0.46	4.01	21.43	62.13	11.97	0.86 **	0.30 **	0.28 **	0.30 **	0.23 **	0.28 **	0.31 **	0.36 **	0.39 **	0.38 **	0.37 **	0.44 **	0.44 **	−0.17 **	−0.11 **	−0.11 **	−0.16 **	−0.13 **	0.57 **	0.51 **	0.57 **	0.61 **	1				
Item23	0.98	6.79	20.56	60.21	11.47	0.80 **	0.25 **	0.26 **	0.27 **	0.24 **	0.25 **	0.27 **	0.31 **	0.32 **	0.31 **	0.34 **	0.43 **	0.50 **	−0.16 **	−0.14 **	−0.16 **	−0.15 **	−0.17 **	0.47 **	0.37 **	0.38 **	0.33 **	0.43 **	1			
Item24	0.81	7.23	23.52	57.96	10.47	0.86 **	0.22 **	0.23 **	0.25 **	0.22 **	0.23 **	0.24 **	0.29 **	0.32 **	0.30 **	0.32 **	0.40 **	0.46 **	−0.18 **	−0.15 **	−0.17 **	−0.17 **	−0.18 **	0.48 **	0.38 **	0.39 **	0.33 **	0.44 **	0.81 **	1		
Item25	0.75	6.6	25.01	58.05	9.58	0.81 **	0.19 **	0.20 **	0.22 **	0.18 **	0.18 **	0.22 **	0.27 **	0.30 **	0.26 **	0.30 **	0.40 **	0.45 **	−0.14 **	−0.11 **	−0.14 **	−0.15 **	−0.13 **	0.49 **	0.36 **	0.37 **	0.33 **	0.44 **	0.72 **	0.8 **	1	
Item26	0.79	5.47	25.17	58.39	10.18	0.81 **	0.20 **	0.22 **	0.24 **	0.23 **	0.22 **	0.27 **	0.30 **	0.33 **	0.30 **	0.31 **	0.43 **	0.44 **	−0.17 **	−0.10 **	−0.12 **	−0.14 **	−0.15 **	0.55 **	0.44 **	0.42 **	0.37 **	0.48 **	0.69 **	0.76 **	0.81 **	1
Whole scale	NA	NA	NA	NA	NA	NA	0.42 **	0.42 **	0.43 **	0.39 **	0.41 **	0.42 **	0.46 **	0.47 **	0.45 **	0.47 **	0.53 **	0.54 **	−0.26 **	−0.23 **	−0.24 **	−0.22 **	−0.26 **	0.56 **	0.55 **	0.56 **	0.49 **	0.55 **	0.60 **	0.57 **	0.55 **	0.57 **

Note: Please refer to Table 1 for the item names and the HBM dimensions. NA = not applicable. ** *p* < 0.01; * *p* < 0.05.
